# Acute Responses in Dynamic Balance, Vertical Jump Performance, and Visuomotor Reaction Time Following Ankle Kinesio Taping and Dynamic Taping in Competitive Basketball Players: A Within-Subject Study

**DOI:** 10.3390/jcm15218427

**Published:** 2026-10-29

**Authors:** Kevser Burma, Meltem Kaya, Gamze Demircioglu

**Affiliations:** 1 Department of Physiotherapy and Rehabilitation, Faculty of Health Sciences, Istanbul Atlas University, Istanbul 34408, Türkiye; meltem.kaya@atlas.edu.tr; 2 Department of Occupational Therapy, Faculty of Health Sciences, Istanbul Atlas University, Istanbul 34408, Türkiye; gamze.demircioglu@atlas.edu.tr

**Keywords:** basketball, dynamic balance, dynamic tape, Kinesio taping, reaction time, vertical jump, Y-Balance Test

## Abstract

**Background/Objectives**: Kinesio taping (KT) and dynamic taping (DT) are commonly used in sports settings to support sensorimotor function and athletic performance. However, evidence directly comparing their immediate effects across different performance domains in competitive basketball players remains limited. This study aimed to compare the acute responses to KT and DT in dynamic balance, vertical jump performance, and visuomotor reaction time. **Methods**: Twenty male competitive basketball players participated in this within-subject study. All participants received KT during the first testing session and DT during the second session conducted one week later. Dynamic balance, vertical jump performance, and visuomotor reaction time were assessed immediately before and after each taping application using the Lower Quarter Y-Balance Test, My Jump 2, and the Light Trainer system, respectively. Within-condition pre–post changes and differences in change between KT and DT were analyzed using paired-samples *t*-tests, with the Holm procedure applied to secondary outcomes to account for multiple comparisons. **Results**: Significant pre–post increases were observed in anterior and posterolateral reach and composite Y-Balance scores following both KT and DT (all *p* < 0.001). Posteromedial reach increased significantly following DT (*p* = 0.003) but not KT (*p* = 0.076), although the between-condition difference in change was not significant. Vertical jump performance and visuomotor reaction time showed no significant pre–post changes following either taping condition. No statistically significant between-condition differences were observed for any outcome. **Conclusions**: Both KT and DT were followed by immediate improvements in dynamic balance, whereas no statistically significant changes were detected in vertical jump performance or visuomotor reaction time. No statistically significant difference in the magnitude of change was observed between the two taping conditions. The differing response patterns across outcomes may indicate task-dependent acute responses; however, this interpretation should be considered preliminary given the study design and sample size.

## 1. Introduction

Basketball requires repeated jumping, sprinting, cutting, and landing actions that place substantial demands on lower-extremity stability, neuromuscular control, and rapid sensorimotor responses [1,2]. Dynamic balance is important for maintaining postural control during complex movements, while vertical jump performance contributes to actions such as rebounding, blocking, and shooting [3]. Visuomotor reaction time is also relevant because players must rapidly process visual information and generate appropriate motor responses in constantly changing game situations [4]. Given the role of the ankle in postural stability and force transmission, strategies aimed at acutely supporting ankle-related sensorimotor function may be relevant to basketball performance [5].

Kinesio taping (KT) is widely used in sports and rehabilitation to provide support without substantially restricting joint motion and has been proposed to enhance proprioceptive feedback and neuromuscular control [6]. Although some studies have reported improvements in balance and functional performance, findings remain inconsistent, particularly for sport-specific outcomes such as vertical jump performance [7,8]. Dynamic taping (DT), in contrast, is based on a different biomechanical rationale and is designed to absorb, store, and release elastic energy during movement while assisting load management [9]. DT may also provide continuous sensory stimulation and has shown beneficial effects on postural control and ankle function, although evidence in competitive athletes remains limited [10].

Despite their different proposed mechanisms, direct comparisons of KT and DT remain scarce. Previous research has largely investigated these approaches separately and has frequently focused on individuals with chronic ankle instability rather than healthy competitive athletes [7,8]. Moreover, although taping may influence peripheral sensory input and sensorimotor integration, its potential effect on visuomotor reaction time has received little attention. To our knowledge, no previous study has directly compared the acute effects of KT and DT across dynamic balance, vertical jump performance, and visuomotor reaction time in competitive basketball players.

Therefore, this study aimed to compare the acute responses to KT and DT applied to the ankle on dynamic balance, vertical jump performance, and visuomotor reaction time in competitive basketball players. We hypothesized that both taping conditions would be associated with acute improvements in the assessed performance outcomes.

## 2. Materials and Methods

### 2.1. Participants

Twenty male competitive basketball players voluntarily participated in the study. Participants were eligible if they were 18–30 years of age, held an active Turkish Basketball Federation license, had at least two years of basketball experience, trained in basketball at least four times per week, and provided written informed consent. Participants were excluded if they had experienced an ankle sprain within the previous six months, had undergone lower-extremity surgery within the previous six months, had a current ankle injury at the time of assessment, or were using medications that could affect balance or motor performance. Limb dominance was determined by self-report and defined as the leg preferentially used to kick a ball.

### 2.2. Study Design and Procedures

This study employed a within-subject design to investigate the acute responses to KT and DT on dynamic balance, vertical jump performance, and visuomotor reaction time in competitive basketball players. All participants completed both taping conditions in a fixed sequence. During the first session, baseline assessments were performed, followed by KT application and immediate post-application assessments. One week later, participants returned for the second session, during which baseline assessments were repeated, followed by DT application and immediate post-application assessments.

Taping procedures and outcome assessments were performed by two different physiotherapists. Taping was applied by a physiotherapist experienced in therapeutic taping, whereas all outcome measurements were conducted by a second physiotherapist who was not involved in the taping procedure. Assessor blinding to the taping condition was not feasible because KT and DT were visually distinguishable during the post-taping assessments. Dynamic balance, vertical jump performance, and visuomotor reaction time were assessed immediately before and after each taping application.

**Figure 1:**
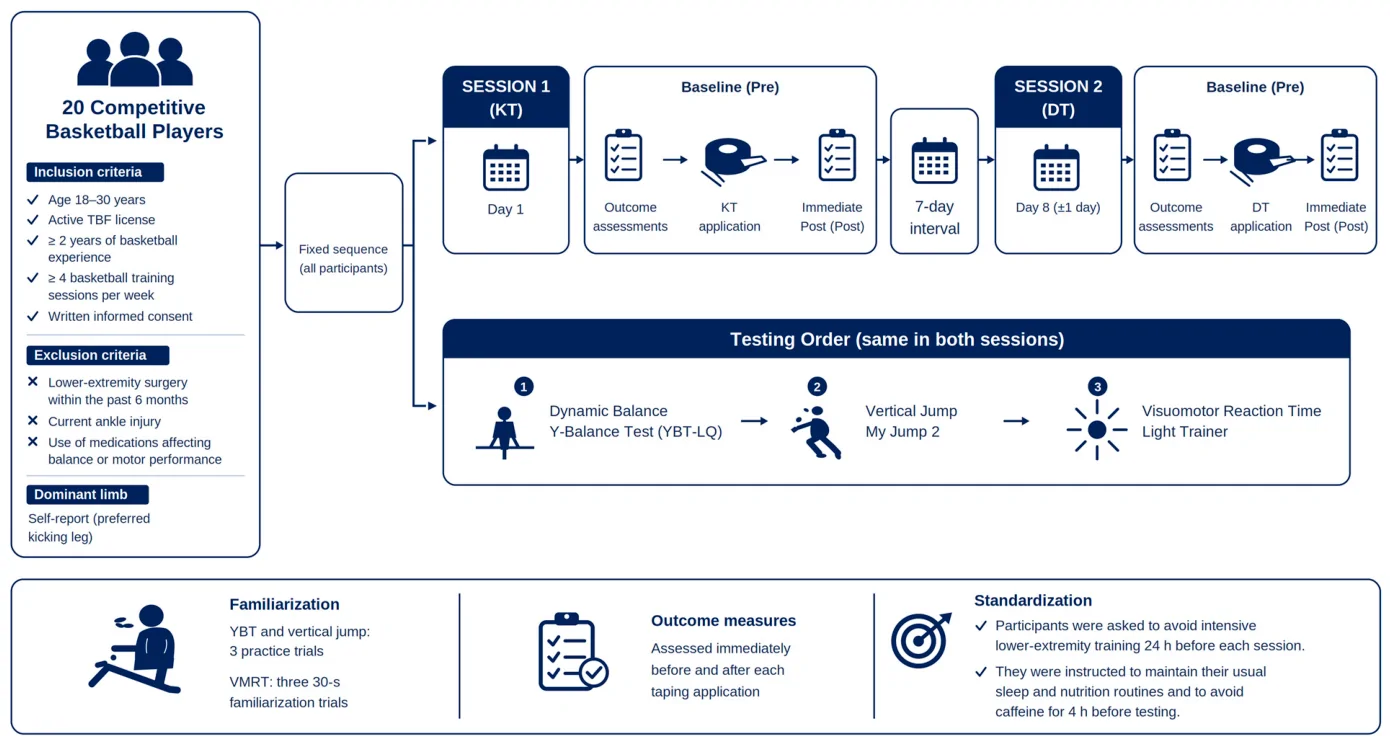
Study design.

Before data collection, participants completed standardized familiarization trials for each performance test to minimize potential learning effects. Three practice trials were performed for the Y-Balance Test and vertical jump test, and three 30-s familiarization trials were completed for the visuomotor reaction time task (Figure 1). To minimize the potential influence of fatigue and recent training load on performance, participants were instructed to avoid vigorous physical activity and strenuous basketball training for 48 h before each testing session. The same testing procedures and test order were maintained across both sessions, and a 2-min rest interval was provided between performance tests.

The study was conducted in accordance with the Declaration of Helsinki and was approved by the Istanbul Medipol University Non-Interventional Clinical Research Ethics Committee (Decision No. 397; 19 February 2026). Written informed consent was obtained from all participants before participation. The study was prospectively registered at ClinicalTrials.gov (number: NCT07404215; date: 5 February 2026).

### 2.3. Outcome Measures

#### 2.3.1. Dynamic Balance

Dynamic balance was assessed using the Lower Quarter Y-Balance Test (YBT-LQ) in the anterior (ANT), posteromedial (PM), and posterolateral (PL) directions. Participants stood barefoot on the dominant limb while maintaining a single-leg stance and reached as far as possible with the contralateral limb in each direction. Three trials were performed in each direction, and the maximum reach distance was recorded. Reach distances were normalized to limb length. The composite score was calculated as the sum of the maximum reach distances in the three directions divided by three times the participant’s limb length and multiplied by 100. The YBT-LQ has demonstrated excellent inter- and intra-rater reliability in healthy adults, with median ICC values ranging from 0.87 to 0.88 and 0.88 to 0.90, respectively [11].

#### 2.3.2. Vertical Jump Performance

Vertical jump performance was assessed using the My Jump 2 mobile application (version 6.1.9; Carlos Balsalobre, Madrid, Spain). Participants performed maximal countermovement jumps with their hands placed on their hips. Three trials were completed, and the highest jump height (cm) was retained for analysis. My Jump 2 has demonstrated excellent reliability and criterion validity for vertical jump assessment, with an ICC of 0.980 and a correlation coefficient of r = 0.990 with reference measures [12].

#### 2.3.3. Visuomotor Reaction Time

Visuomotor reaction time was assessed using a Light Trainer system (Light TrainerVisuo-motor Equipments Company, Istanbul, Türkiye) consisting of eight wireless light modules. Participants maintained single-leg stance on the dominant limb and responded as quickly as possible to randomly illuminated targets using the contralateral lower extremity. Before testing, participants completed three 30-s familiarization trials, with 30-s rest intervals between trials. Following familiarization, a 1-min test trial was performed. Mean reaction time (s) was recorded and used for statistical analysis. The testing procedure was based on a previously described lower-extremity protocol using a light-based reaction system, which demonstrated good-to-excellent test–retest reliability in healthy, physically active adults (ICC = 0.80–0.92) [4]. Similar light-based lower-extremity reaction-time assessments have also been used in athletic populations, including football and basketball players [13,14].

### 2.4. Taping Procedures

**Figure 2:**
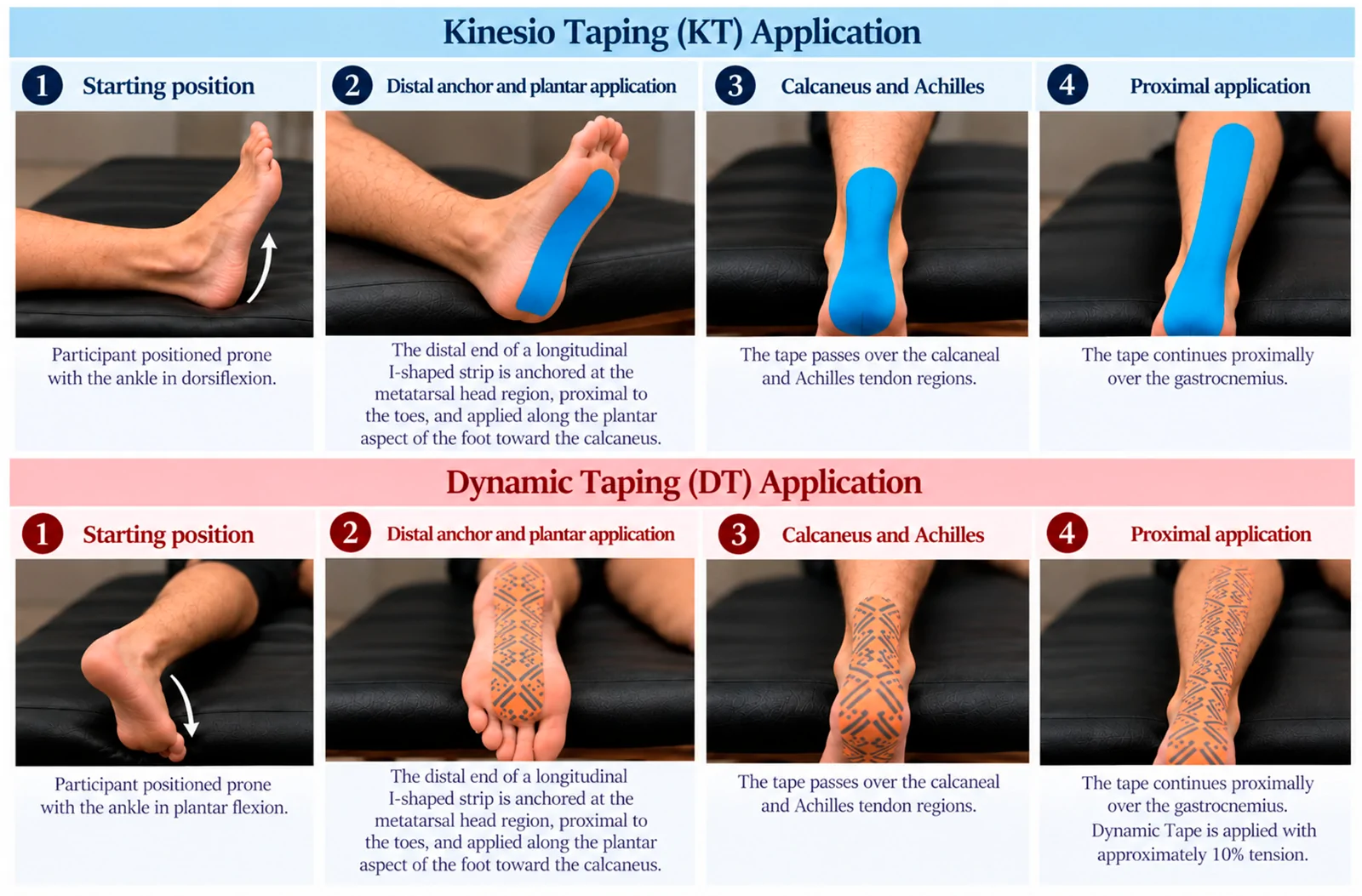
Kinesio Taping (KT) and Dynamic Taping (DT) Application Procedures.

Both taping applications were performed by the same physiotherapist following standardized procedures based on previously described taping principles [9,10,15] (Figure 2). Before application, the skin was cleaned and dried, and participants were positioned prone. For both conditions, a longitudinal I-shaped strip was applied from the metatarsal head region along the plantar aspect of the foot, passing over the calcaneal and Achilles tendon regions and continuing proximally over the gastrocnemius.

For Kinesio Taping (KT) (Cre-Med, Albuquerque, NM, USA), the ankle was positioned in dorsiflexion, consistent with previously described kinesiology taping procedures [15]. For Dynamic Taping (DT) (PosturePals Pty Ltd, Port Vila, Vanuatu), the ankle was positioned in plantar flexion, consistent with previously described Dynamic Taping principles [9,10]. Dynamic Tape was applied with approximately 10% tension, consistent with previously described application protocols [15]. Thus, although both applications followed a comparable anatomical pathway, ankle positioning and tape-specific application principles differed between KT and DT.

Participants were assessed immediately before and after each taping application to examine the acute responses following taping.

### 2.5. Sample Size Calculation

An a priori sample size calculation was performed using G*Power software (version 3.1.9.7; Heinrich Heine University, Düsseldorf, Germany) based on the primary outcome, the Y-Balance Test composite score. Based on previous studies demonstrating changes in postural control following kinesio and dynamic taping applications [10,16], a large within-subject effect size (dz = 0.80) was assumed. With a two-tailed significance level of 0.05 and a statistical power of 80%, the minimum required sample size was calculated as 15 participants. To account for potential dropouts or incomplete data, 20 participants were recruited.

### 2.6. Statistical Analysis

Statistical analyses were performed using IBM SPSS Statistics for Windows, version 29.0 (IBM Corp., Armonk, NY, USA). The normality of continuous variables and within-subject difference scores was assessed using the Shapiro–Wilk test. Descriptive data are presented as mean ± standard deviation (SD). The primary outcome was the Y-Balance Test composite score, while anterior, posteromedial, and posterolateral reach scores, vertical jump height, and visuomotor reaction time were considered secondary outcomes. To account for multiplicity among the secondary outcomes, the Holm procedure was applied separately within each analysis family: KT pre–post comparisons, DT pre–post comparisons, and between-condition comparisons of change scores. The primary outcome was analyzed separately and was not included in the multiplicity adjustment. Within each taping condition, pre- and post-application values were compared using paired-samples *t*-tests. To compare the magnitude of acute responses between KT and DT, change scores (Δ) were calculated as post-application minus pre-application values for each condition and subsequently compared using paired-samples *t*-tests. Mean differences with 95% confidence intervals were reported where appropriate. Effect sizes for paired comparisons were calculated using Cohen’s dz, defined as the mean paired difference divided by the standard deviation of the paired differences. Effect sizes were calculated for both within-condition pre–post changes and between-condition comparisons of change scores. Effect sizes were interpreted based on their absolute magnitude as negligible (<0.20), small (0.20–0.49), moderate (0.50–0.79), or large (≥0.80). Statistical significance was set at *p* < 0.05.

## 3. Results

Twenty male competitive basketball players completed both testing sessions and were included in the final analysis. No participants withdrew from the study or had incomplete data. No adverse events or skin reactions were reported during either taping condition. Participant characteristics are presented in Table 1.

Following KT application, significant increases were observed in anterior reach (*p* < 0.001), posterolateral reach (*p* < 0.001), and the Y-Balance composite score (*p* < 0.001), whereas changes in posteromedial reach (*p* = 0.076), vertical jump height (*p* = 0.135), and visuomotor reaction time (*p* = 0.246) were not statistically significant. Following DT application, significant increases were observed in anterior reach (*p* < 0.001), posteromedial reach (*p* = 0.003), posterolateral reach (*p* < 0.001), and the Y-Balance composite score (*p* < 0.001), whereas no statistically significant changes were observed in vertical jump height (*p* = 0.095) or visuomotor reaction time (*p* = 0.569).

When change scores were compared between KT and DT, no statistically significant between-condition differences were detected for vertical jump height (*p* = 0.615), anterior reach (*p* = 0.930), posteromedial reach (*p* = 0.365), posterolateral reach (*p* = 0.297), the Y-Balance composite score (*p* = 0.932), or visuomotor reaction time (*p* = 0.767) (Table 2).

Effect-size estimates showed large within-condition effects following KT for anterior reach (Cohen’s dz = 1.61), posterolateral reach (dz = 3.05), and the Y-Balance composite score (dz = 2.61), with a moderate effect for posteromedial reach (dz = 0.51) and small effects for vertical jump height (dz = 0.43) and visuomotor reaction time (|dz| = 0.32). Following DT, large effects were observed for anterior reach (dz = 1.15), posteromedial reach (dz = 0.97), posterolateral reach (dz = 1.71), and the Y-Balance composite score (dz = 2.81), whereas the effects were small for vertical jump height (dz = 0.48) and negligible for visuomotor reaction time (|dz| = 0.16). Between-condition effect sizes for the differences in change scores were negligible to small across all outcomes (|dz| = 0.02–0.29).

## 4. Discussion

The present within-subject study examined the acute responses to kinesio taping (KT) and dynamic taping (DT) across three performance domains relevant to basketball: dynamic balance, vertical jump performance, and visuomotor reaction time. Both taping conditions were followed by significant improvements in anterior and posterolateral reach and composite Y-Balance scores, whereas posteromedial reach improved significantly only following DT. In contrast, neither vertical jump performance nor visuomotor reaction time changed significantly following either taping condition. Importantly, no statistically significant differences in the magnitude of change were detected between KT and DT for any outcome. Taken together, these findings suggest that the immediate responses observed following ankle taping may be task-specific, with greater relevance to dynamic postural control than to explosive or visuomotor performance. The improvements observed in dynamic balance following both taping conditions may be related to increased sensory input and enhanced sensorimotor control. Cutaneous stimulation produced by taping has been proposed to increase afferent feedback from mechanoreceptors and thereby contribute to joint position awareness and postural regulation [16,17]. Such sensory input may be particularly relevant during the Y-Balance Test, which requires continuous integration of proprioceptive information, single-leg postural control, and coordinated lower-extremity movement. Consistent with this concept, Tedeschi et al. [18] demonstrated the importance of sensory information for postural control during single-limb stance, particularly when visual input was removed. Their findings also showed that balance performance was influenced by age and testing condition, whereas no significant effects of limb dominance or sex were observed. The postural and sensorimotor demands of the Y-Balance Test are also relevant to several basketball-specific actions, including landing, cutting, and rapid changes of direction, during which athletes must continuously regulate their center of mass over a changing base of support. Previous studies have similarly reported improvements in balance and proprioceptive performance following elastic taping applications in athletic and ankle-instability populations [19,20,21]. Although posteromedial reach increased significantly following DT but not KT, this finding should be interpreted cautiously. The magnitude of change in posteromedial reach did not differ significantly between the two taping conditions, indicating that statistical significance within one condition but not the other does not constitute evidence of a differential treatment response. Accordingly, the present data do not support a superior effect of DT on posteromedial reach. The direction-specific difference may instead reflect variability in within-subject responses or the specific biomechanical demands of the posteromedial reach task. Larger studies specifically powered to examine directional Y-Balance outcomes would be needed to determine whether this observation represents a consistent response pattern. The between-condition analyses in the present study showed no significant differences in any Y-Balance outcome. Although statistically significant improvements with large within-condition effect sizes were observed in Y-Balance performance, the clinical relevance of these changes should be interpreted cautiously. Previous reliability studies have reported measurement error and minimal detectable change (MDC) values for Y-Balance outcomes; however, these estimates vary according to the population, reach direction, and testing protocol. As population-specific measurement error and MDC values were not established in the present sample of competitive basketball players, it cannot be determined with certainty whether the observed changes exceeded measurement error. Furthermore, a population-specific minimal clinically important difference has not been established; therefore, statistical significance and large effect sizes should not be interpreted as evidence of clinically meaningful improvement.

Despite their partly different theoretical mechanisms, KT and DT were associated with similar patterns of acute response in the present study. KT is primarily intended to augment cutaneous and proprioceptive input while providing support without substantially restricting joint motion, whereas DT additionally emphasizes load absorption, elastic energy storage and return, and mechanical assistance during movement [6,9]. These mechanistic distinctions did not translate into statistically detectable differences in the measured performance outcomes. One possible explanation is that the sensory input common to both taping approaches may be more relevant to a relatively controlled dynamic balance task than their distinct mechanical characteristics. Alternatively, the biomechanical advantages proposed for DT may become more apparent during tasks involving greater external loads, repeated stretch-shortening cycles, or prolonged movement rather than during the immediate performance assessments used in the present study. Nevertheless, given the sample size and fixed intervention sequence, the absence of statistically significant differences should not be interpreted as evidence that the two taping methods are equivalent [11].

In contrast to dynamic balance, vertical jump performance did not change significantly following either KT or DT. Vertical jumping is a complex explosive task that depends on rapid force production, lower-extremity muscle strength, stretch-shortening cycle efficiency, and intersegmental coordination. Acute changes in cutaneous afferent input or proprioceptive awareness may therefore be insufficient to produce measurable changes in the neuromuscular power required for maximal jumping. This interpretation is consistent with previous research showing inconsistent or limited effects of taping on vertical jump performance [6,16,22,23]. In addition, the participants in the present study were licensed competitive basketball players who trained regularly, and their established neuromuscular adaptations may have reduced the potential for additional immediate improvement. However, because no direct measure of maximal strength or lower-extremity power reserve was obtained, a ceiling effect cannot be confirmed. Moreover, the absence of a statistically significant change in vertical jump performance should not be interpreted as evidence that taping has no acute effect on this outcome, as the study may have been insufficiently powered to detect smaller changes and the measurement approach may not have been sensitive to subtle acute responses.

Visuomotor reaction time also remained unchanged following both taping conditions. Unlike dynamic balance, visuomotor reaction performance requires rapid visual stimulus detection, central sensorimotor integration, response selection, and subsequent motor execution [24]. Although ankle taping may modify peripheral somatosensory input, such changes may not be sufficient to influence the broader neural processes underlying visuomotor responding. This may explain why improvements in dynamic balance were not accompanied by faster reaction responses. Therefore, the different response patterns observed across the Y-Balance Test, vertical jump, and visuomotor reaction time may indicate task-dependent acute responses; however, this interpretation should be considered preliminary. Improvements in Y-Balance performance may have been influenced by repeated testing or learning, whereas the absence of statistically significant changes in vertical jump performance and visuomotor reaction time may also reflect insufficient statistical power or limited measurement sensitivity rather than genuine task specificity.

From a practical perspective, the present findings suggest that KT and DT may have potential as short-term adjunctive strategies when the primary objective is to support dynamic balance. However, no statistically significant acute improvements in vertical jump performance or visuomotor reaction time were observed following either taping condition. These findings may be relevant to clinicians and strength and conditioning professionals when selecting taping interventions according to the specific performance demand being targeted. Rather than considering taping as a general performance-enhancing strategy, its immediate utility may be more appropriately viewed as task-dependent and primarily related to sensorimotor and postural functions. Nevertheless, because the study did not include a no-tape or sham-taping condition, the observed pre–post improvements cannot be attributed exclusively to the taping applications.

Several limitations should be considered when interpreting these findings. First, only immediate responses to KT and DT were investigated; therefore, the effects of repeated applications or longer-term use remain unknown. In addition, proprioception, muscle activity, joint mechanics, and load modification were not directly measured; therefore, the sensory and biomechanical mechanisms proposed to explain the observed responses should be regarded as theoretical interpretations rather than directly demonstrated mechanisms. Second, the relatively small and homogeneous sample limits the generalizability of the findings. In addition, the priori sample-size calculation was based on detecting a large within-subject change in the primary outcome and was not specifically powered for the between-condition comparison of change scores. Therefore, the absence of statistically significant differences between KT and DT should not be interpreted as evidence of equivalence. Third, the study did not include a sham-taping or no-taping control condition, the observed pre–post changes cannot be attributed specifically to either taping intervention, as repeated testing, familiarization, expectancy, or other within-session factors may also have contributed to the observed responses. Future studies incorporating sham or no-taping control conditions are required to determine whether these changes are specifically attributable to the taping interventions. KT was consistently applied during the first session and DT during the second session, without randomization or counterbalancing. Therefore, the observed responses cannot be fully separated from potential order, period, learning, or repeated-testing effects. Although standardized familiarization trials were performed before data collection, repeated exposure, particularly to the Y-Balance Test, may still have influenced performance. Future studies should therefore employ randomized and counterbalanced intervention sequences to better distinguish intervention-related responses from order and repeated-testing effects.

## 5. Conclusions

Both kinesio taping and dynamic taping were followed by acute improvements in dynamic balance in competitive basketball players, whereas no statistically significant changes were detected in vertical jump performance or visuomotor reaction time. No statistically significant differences in the magnitude of change were observed between the two taping conditions. The differing response patterns across outcomes may indicate task-dependent acute responses; however, this interpretation should be considered preliminary given the study design and sample size. Further randomized studies including sham or no-tape control conditions are needed to confirm these findings and clarify the relative effects of different taping approaches.

## Figures and Tables

**Table 1:** Demographic and Physical Characteristics of Participants.

Variable	Mean ± SD
Age (years)	28.00 ± 1.36
Height (cm)	197.14 ± 2.38
Body mass (kg)	96.29 ± 2.56
BMI (kg/m^2^)	24.64 ± 0.40
Leg length (cm)	101.93 ± 1.69
Shoe size	47.14 ± 0.37

SD = standard deviation.

**Table 2:** Pre- and Post-Application Outcomes and Comparison of Change Scores Between Kinesio and Dynamic Taping Conditions.

Variable	KT Pre (Mean ± SD)	KT Post (Mean ± SD)	KT Δ (Mean ± SD)	*p*	Cohen’s dz	DT Pre (Mean ± SD)	DT Post (Mean ± SD)	DT Δ (Mean ± SD)	*p*	Cohen’s dz	Mean Δ Difference (KT − DT) [95% CI]	Δ *p* (KT vs. DT)	KT vs. DT Cohen’s dz
Vertical jump height (cm)	26.54 ± 3.78	27.61 ± 4.11	1.08 ± 2.53	0.135	0.43	26.94 ± 3.55	28.34 ± 3.11	1.40 ± 2.91	0.095	0.48	−0.32 [−1.67, 1.03]	0.615	0.14
Anterior reach distance (%)	74.29 ± 4.51	76.93 ± 4.84	2.64 ± 1.65	<0.001	1.61	74.86 ± 5.42	77.57 ± 6.28	2.71 ± 2.37	<0.001	1.15	−0.07 [−1.79, 1.65]	0.930	0.02
Posteromedial reach distance (%)	103.64 ± 3.89	105.43 ± 5.27	1.79 ± 3.47	0.076	0.51	103.86 ± 4.72	106.50 ± 4.00	2.64 ± 2.73	0.003	0.97	−0.86 [−2.83, 1.12]	0.365	0.25
Posterolateral reach distance (%)	95.07 ± 3.91	101.93 ± 5.03	6.86 ± 2.25	<0.001	3.05	96.43 ± 4.24	102.29 ± 5.69	5.86 ± 3.42	<0.001	1.71	1.00 [−0.99, 2.99]	0.297	0.29
Composite score (%)	89.41 ± 2.93	93.10 ± 3.47	3.69 ± 1.42	<0.001	2.61	90.11 ± 2.99	93.77 ± 3.39	3.66 ± 1.30	<0.001	2.81	0.03 [−0.75, 0.81]	0.932	0.02
Mean reaction time (s)	0.40 ± 0.06	0.38 ± 0.03	−0.02 ± 0.07	0.246	0.32	0.40 ± 0.06	0.39 ± 0.05	−0.01 ± 0.09	0.569	0.16	−0.009 [−0.075, 0.057]	0.767	0.08

KT = kinesio taping; DT = dynamic taping; SD = standard deviation; CI = confidence interval; Δ = change from pre- to post-application. Mean Δ Difference represents the mean paired difference in change scores between KT and DT (KT − DT), presented with its 95% CI. Δp (KT vs. DT) refers to the paired-samples *t*-test comparison of change scores between conditions. Cohen’s dz represents the standardized effect size for paired comparisons and was calculated as the mean paired difference divided by the standard deviation of the paired differences. Reported *p* values are unadjusted; for secondary outcomes, statistical significance was evaluated after adjustment for multiple comparisons using the Holm procedure, applied separately within the KT pre–post, DT pre–post, and between-condition change-score comparison families. The Y-Balance Test composite score was the primary outcome and was not included in the multiplicity adjustment.

## Data Availability

The data presented in this study are available from the corresponding author upon reasonable request. The data are not publicly available due to privacy and ethical considerations related to human participants.
